# P-1908. It's All in Your Head (Mounted Display): Early Findings from Virtual Reality Training for Infection Prevention and Control

**DOI:** 10.1093/ofid/ofaf695.2077

**Published:** 2026-01-11

**Authors:** Michelle S Jerry, Vianelly García, Chloe V Green, Andrea S Greenfield, Eileen F Searle, Erica S Shenoy

**Affiliations:** Massachusetts General Hospital, Boston, Massachusetts; Massachusetts General Hospital, Boston, Massachusetts; Massachusetts General Hospital, Boston, Massachusetts; Massachusetts General Hospital, Boston, Massachusetts; Massachusetts General Hospital, Boston, Massachusetts; Mass General Brigham, Boston, MA

## Abstract

**Background:**

Portable Medical Equipment (PME) is often highly contaminated, risking transmission of healthcare-associated infections (HAI); contributing factors include failures by healthcare personnel (HCP) to correctly and consistently clean and disinfect devices. Emerging evidence highlights Virtual Reality (VR) as a promising tool for training HCP; however, objective measures of effectiveness are needed.

**Figure 1.**
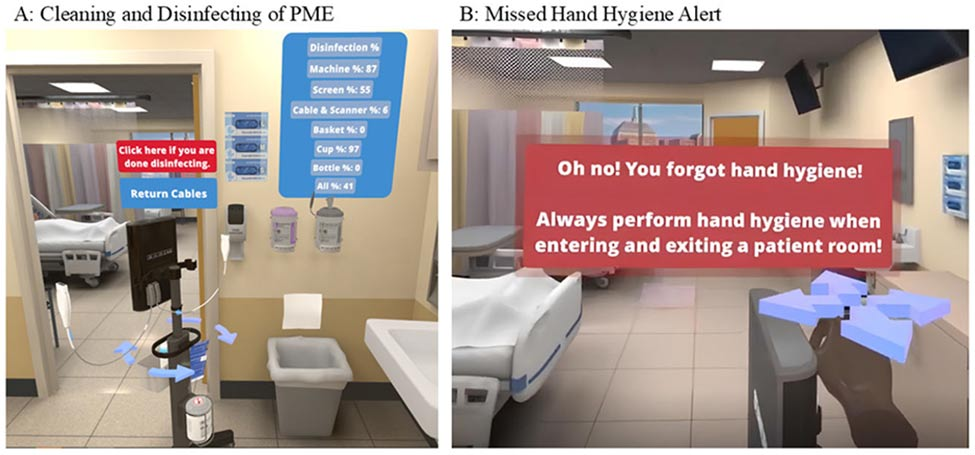
Percentage Disinfected of PME (A) and Hand Hygiene Alert (B). In panel A, after participants have completed cleaning any visible soil (e.g., ultrasound gel) on the equipment, they are prompted to begin disinfecting. Their performance is displayed as the percentage of the machine that was disinfected in real time. In panel B, if the participant tries to enter or exit the patient room without performing hand hygiene (either with soap and water or alcohol-based hand rub), they are alerted of the missed step and their performance recorded.

**Figure 2.**
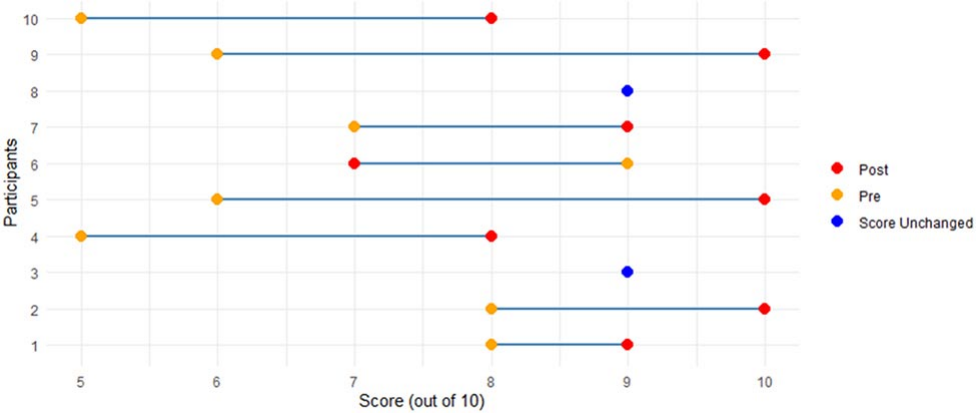
Paired Knowledge Scores Pre vs. Post by Participant

**Methods:**

A VR module was created by experts in infection prevention and control (IPC) and competency-based education to train HCP on cleaning and low-level disinfection of PME, such as a vital signs or point-of-care ultrasound machine. Performance metrics captured by the headset included the percentage of PME disinfected and hand hygiene compliance. Participants completed pre- and post-intervention surveys assessing knowledge, skills, and attitudes (KSA). Participant experience was assessed as part of an early phase pilot study.

**Figure 3.**
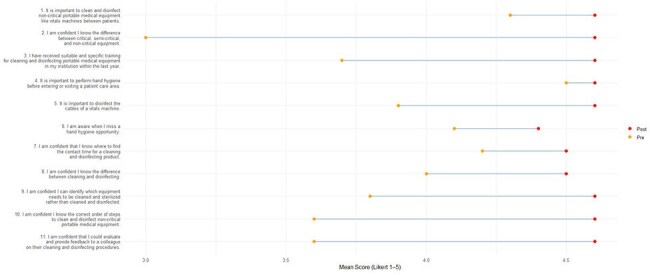
Mean Pre vs Post IPC Attitude Question Likert Scores

**Results:**

In April 2025, 10 participants completed the VR training module. The majority of participants were Registered Nurses (n=8, 80%), between the ages of 30 and 54, who had either never tried VR before (n=4, 40%), or who had only tried it one time (n=6, 60%). Performance metrics captured by the headset demonstrated that participants disinfected 40 to 100% of the components of the PME and all but one participant missed hand hygiene at least once during the module (Figure 1). Compared to pre-test mean results, participants showed an increase in IPC knowledge (Figure 2) and increased confidence in cleaning and disinfecting principles (Figure 3). 90% of users also agreed or strongly agreed that the module was more engaging compared to previous IPC training programs.

**Conclusion:**

VR has the potential to bridge the gap between traditional training modalities, which rely on abstract concepts in classroom settings, and real-world application. Though limited in size, this study demonstrated an increase in user knowledge and confidence in implementing IPC guidelines to HCP clinical workflows through use of VR and supports efforts to implement larger-scale studies to assess effectiveness.

**Disclosures:**

Erica S. Shenoy, MD, PhD, Up to Date: Author

